# Model-Independent Phenotyping of *C. elegans* Locomotion Using Scale-Invariant Feature Transform

**DOI:** 10.1371/journal.pone.0122326

**Published:** 2015-03-27

**Authors:** Yelena Koren, Raphael Sznitman, Paulo E. Arratia, Christopher Carls, Predrag Krajacic, André E. X. Brown, Josué Sznitman

**Affiliations:** 1 Department of Biomedical Engineering, Technion—Israel Institute of Technology, Israel; 2 Ophthalmic Technology Group, ARTORG Center, University of Bern, Switzerland; 3 Department of Mechanical Engineering and Applied Mechanics, University of Pennsylvania, Philadelphia PA, USA; 4 Department of Biomedical Sciences, West Virginia School of Osteopathic Medicine, Lewisburg WV, USA; 5 MRC Clinical Sciences Centre, Faculty of Medicine, Imperial College London, UK; Inserm U869, FRANCE

## Abstract

To uncover the genetic basis of behavioral traits in the model organism *C. elegans*, a common strategy is to study locomotion defects in mutants. Despite efforts to introduce (semi-)automated phenotyping strategies, current methods overwhelmingly depend on worm-specific features that must be hand-crafted and as such are not generalizable for phenotyping motility in other animal models. Hence, there is an ongoing need for robust algorithms that can automatically analyze and classify motility phenotypes quantitatively. To this end, we have developed a fully-automated approach to characterize *C. elegans*’ phenotypes that does not require the definition of nematode-specific features. Rather, we make use of the popular computer vision Scale-Invariant Feature Transform (SIFT) from which we construct histograms of commonly-observed SIFT features to represent nematode motility. We first evaluated our method on a synthetic dataset simulating a range of nematode crawling gaits. Next, we evaluated our algorithm on two distinct datasets of crawling *C. elegans* with mutants affecting neuromuscular structure and function. Not only is our algorithm able to detect differences between strains, results capture similarities in locomotory phenotypes that lead to clustering that is consistent with expectations based on genetic relationships. Our proposed approach generalizes directly and should be applicable to other animal models. Such applicability holds promise for computational ethology as more groups collect high-resolution image data of animal behavior.

## Introduction

*Caenorhabditis elegans* (*C. elegans*) is perhaps the best understood metazoan in terms of anatomy, genetics, development, and behaviour [1]. This transparent, free-living nematode has been widely used as a model organism ever since its first introduction over 40 years ago [2]. In particular, *C. elegans* is commonly used to investigate fundamental questions in biology, including behavioural genetics [3], neuroscience [4, 5], drug screening and development [6] and modelling different aspects of human diseases amongst others [7].

To uncover the genetic basis of behavioural traits in *C. elegans*, a widespread strategy has been to study experimentally locomotion defects in mutants. In reverse genetics, strains with known mutations are phenotyped to determine whether or not the gene of interest has an effect on motility [8]. Since traditional approaches to classifying patterns of *C. elegans* movement have often been based on manual annotation [9], motility phenotyping is often imprecise or qualitative, as well as time consuming. As a result, there is a necessity for algorithms that can automatically analyse and classify *C. elegans* motility phenotypes quantitatively.

In general, (semi-)automated classification methodologies developed to date have been centred around the combination of (i) computer vision techniques to extract motility data from images [10–24] and (ii) statistical learning techniques to build classifiers of observed motility data [10–14, 16, 22, 23, 25, 26]. The first step typically includes the extraction of motility data by segmenting nematodes from their backgrounds (*i.e*. environment) in video sequences; this step has frequently been accompanied by the extraction of the nematode centreline (*i.e*. “skeleton”) in order to provide a one-dimensional line representation of nematode kinematics. Specific features describing physical properties such as body length, speed, angular position, body texture (*i.e*. gray-scale intensity) or posture (*i.e*. curvature) to name but a few, are then extracted either directly from the segmented binary worm image or the extracted skeleton [10, 11, 14, 18].

Studies on *C. elegans* locomotion in various environments (*e.g*. fluid) have coupled body kinematics extracted from digital image processing with biomechanical and hydrodynamic models from which parameters such as nematode tissue stiffness (*e.g*. Young’s modulus), propulsive forces, power (*i.e*. thrust) and tissue viscosity have been estimated [17–21, 24]. The use of such biomechanical motility parameters, coined biomechanical profiling (BMP), was recently introduced to analyse the phenotypic properties of several well-described mutants with defects in neuromuscular structure and function [22], in an effort to deliver more sensitive metrics for these defects. Quantitative BMP parameters were then clustered using standard hierarchical clustering techniques [27]. Parallel to these efforts, an alternative phenotyping approach suggested using an unsupervised search for behavioural motifs to define locomotive phenotypes [23]. In their work, the authors projected nematode skeletons onto wild-type-derived “eigenworms” corresponding to four basic body postures previously described for wild-type nematodes [28] and subsequently searched for closely repeated subsequences. The motifs were combined into a dictionary and used to quantitatively relate mutants to each other [23].

All the aforementioned phenotyping methods are based on initially segmenting worms from their background (environment) and subsequently deriving worm-specific morphological and kinematic features. These frameworks have the advantage of having physically interpretable features; these however are time consuming to define and do not generalise to animals with different morphologies or interacting animals. Moreover, the misclassification error rate may rise significantly when attempting to classify mutants with closely-related phenotypes [14]. While up to several hundred features may be “hand-crafted” at first, dimensionality reduction (*e.g*. PCA) has revealed that not all of these features are informative and that the category variance can be captured by a much smaller subset of features [10, 11, 25].

Motivated by these ongoing limitations, we have developed a novel approach to characterize *C. elegans* phenotypes that does not require the definition of animal-specific features. Instead, we make use of the widely-known Scale-Invariant Feature Transform (SIFT) [29] as an elementary image feature from which we construct histograms of commonly-observed SIFT features to represent nematode motility; hence, our approach is entirely independent of any physical parameters characterizing the nematode. We first evaluated our proof-of-concept method on a synthetic dataset simulating a range of idealized, yet distinct *C. elegans* locomotory behaviours [30]. Next, we evaluated our SIFT-based approach on two distinct datasets of crawling *C. elegans* with mutants affecting neuromuscular structure and function; namely, (i) a subset of 15 mutant strains from a database of single-worm tracking videos [25] and (ii) a set of 8 strains analysed using the recent BMP method [22]. Overall, our proposed approach generalises directly and should be applicable to other animals, even those with very different morphologies, with little foreseen modification to the general algorithmic framework. Applicability across other animal models is important for realizing the full promise of computational ethology as more groups collect high-resolution image data of animal behaviour [31].

## Results

The following results were obtained using our SIFT-BoW algorithm (see Methods section). Briefly, our method characterizes videos of visible motility features by constructing a visual vocabulary of nematode motion. Our vocabulary relies on visual “words” (or image features with large description power), and allows us to construct histograms of occurring words to describe any video sequence. These histograms can then be compared to each other providing a quantitative similarity metric for locomotion. See Methods for more details and Supplementary Information (S1 Matlab) for available open source Matlab code.

### Simulated datasets

In order to assess the feasibility of our phenotyping algorithm, we first evaluated it on a synthetically generated dataset simulating a range of ideal, yet distinctive, *C. elegans* locomotory behaviours. The motivation for investigating synthetic nematode locomotion is that we can quantify how physical parameters affecting locomotory phenotypes (*e.g*. body amplitude, beating frequency, body wavelength) are mapped by our approach; this first step seems appropriate given the wide variability of *C. elegans* motile behaviour under crawling assays [32].

Briefly, the spatio-temporal kinematics of a nematode are modelled as a sinusoidal travelling wave (see S2 Script for open source Matlab script), *i.e*. *A* sin (*kx* − *ωt*)*e* ^−*x*/*l*^, where *A* is the nematode body amplitude, *k* is the wavenumber (*k* = 2*π*/*λ*, where *λ* is the nematode wavelength), *ω* is the nematode’s angular beating frequency (*ω* = 2*πf*, where *f* is the frequency in Hz), *x* represents a vector of spatial coordinates (∼ 200 pixels long) and *l* is the exponential decay length of the nematode body amplitude from head to tail. The nematode forward speed is fixed by *U* = *ω*/*k* such that the worm does not appear to slide but delivers a smooth crawling gait. Note that the modelled kinematics are restricted to the main locomotory behaviour of *C. elegans* crawling forward [18, 21, 30]; such motion represents only a subset of the wide range of known behaviours exhibited by the nematode [25, 33, 34], including amongst other moving backwards, pauses, deep bends, or reversals and reorientations known as “omega turns”.

**Fig 1:**
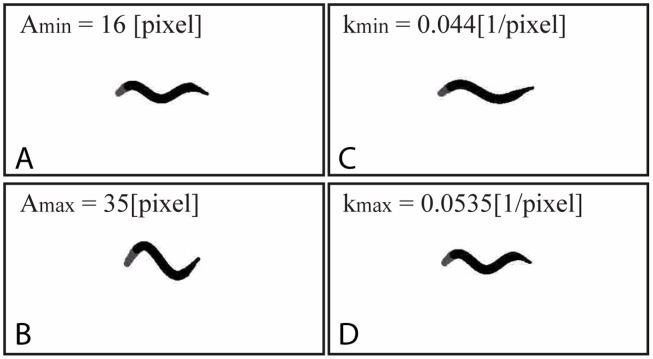
Instantaneous snapshots of synthetically-generated nematode postures. Sample postures shown for the range of (A) minimum and (B) maximum body amplitudes *A* as well as (C) minimum and (D) maximum body wavenumber *k*, according to Table 1. Corresponding supplementary videos are available in the SM.

To test our algorithm, we generated 3 distinct datasets where each set consists of 20 simulated strains, with 20 individual videos in every strain. For each dataset, one of the main parameters (*i.e*. body amplitude, beating frequency or body wavelength) was gradually changed across the simulated strains, while the other two parameters were held constant (see Table 1). Values of the parameters were chosen to match realistic physiological properties of crawling *C.elegans* [30]. In particular, to model the variability of nematode motility behaviour [30], a finite amount of noise was included in the synthetic datasets, where videos within a given strain include a 10% variance for each parameter. Each simulated video represents 10 seconds of motion, constructed of 250 individual frames acquired at 25 frames per second (fps); note that the background of the videos is white without texture or noise. Moreover, similar to recent single-worm tracking experiments for crawling assays [23, 25], worms are re-entered in the frame based on the skeleton centroid position. Examples of nematode body postures are presented in Fig. 1 for sample combinations of the controlled locomotory parameters (see Supplementary Information for corresponding movies S1-S6 of simulated worms).

**Table 1:** Parameter values and corresponding units for simulated datasets of synthetic nematodes. For each parameter value a 10% variance is included to account for variability in nematode motility behaviour according to published data [30].

Simulated Set	Amplitude *A* [pixel]	Frequency *f* [Hz]	Wavenumber *k* = 2*π*/*λ* [1/pixel]
I	16: 1: 35	0.36	0.05
II	18	0.06: 0.02: 0.44	0.05
III	18	0.36	0.044: 5 × 10^−4^: 0.0535

**Fig 2:**
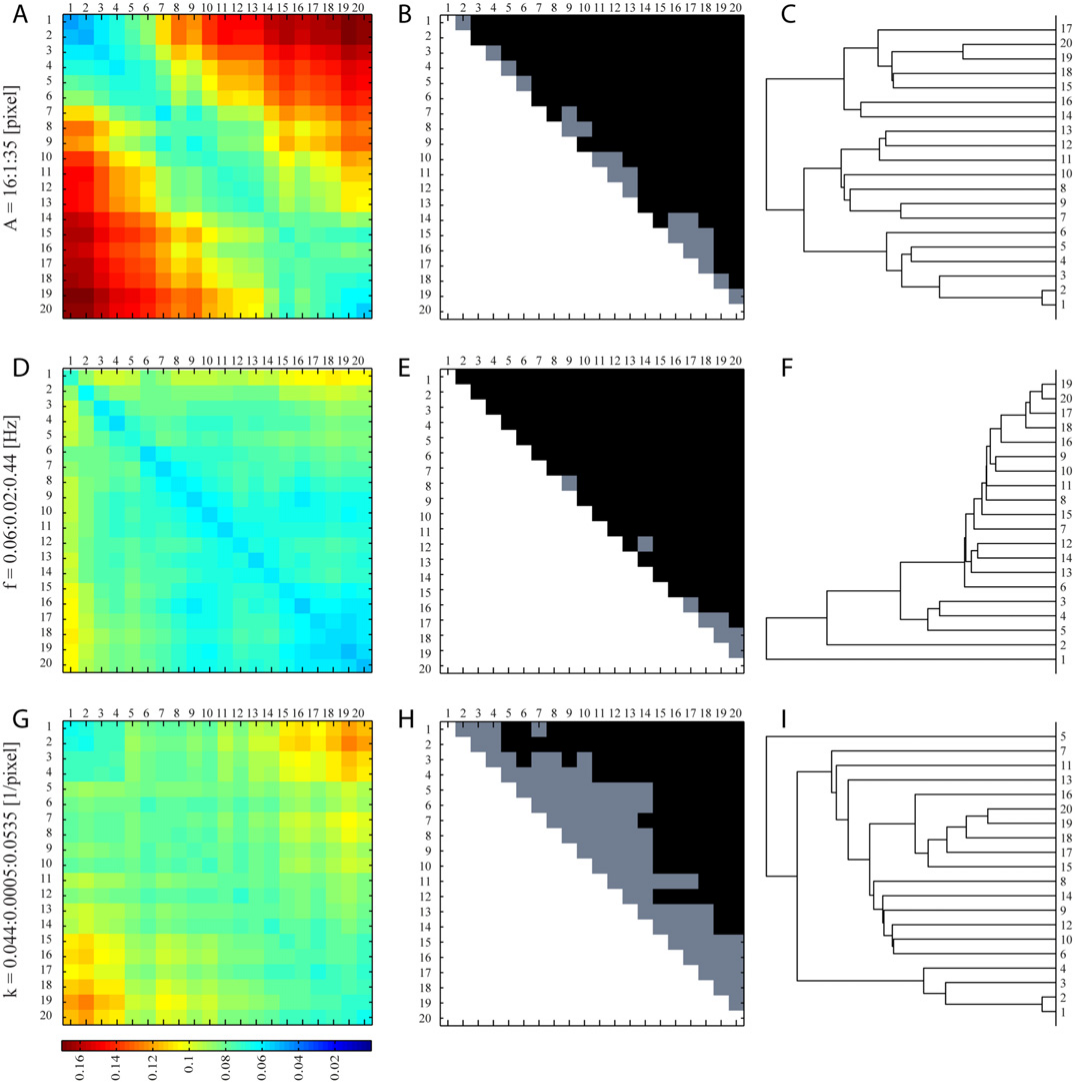
Phenotyping analysis for synthetic nematode data (range shown in Table 1). Left column: distance matrix of average Euclidean distances between classes for parameter sweep in amplitude *A* (A), body frequency *f* (D) and body wavenumber *k* (G). Middle column (B, E, H): corresponding matrix for *p*-values obtained when comparing pairwise a given class with another, following a non-parametric two-sample test for multivariate samples using the minimum statistical energy test [35]. Note that matrices only show values in the upper-triangular region (due to symmetry) where the diagonal is not computed. Significance (black tiles) is set for a confidence level of 95% (*p* < 0.05). Right column (C, F, I): corresponding branching diagrams (*i.e*. dendrograms) that represent a hierarchy based on the relationships of similarity among different classes.

In Fig. 2 (left column), we present the average distance matrices for the three simulated datasets of Table 1. Matrices are symmetric along their diagonal line and color-coded according to the mean Euclidean pairwise distances (in arbitrary units) between simulated strains; each coloured tile in the map represents the average distance for 20 × 20 pairs. Across the three matrices a rather striking pattern arises; this is most distinguishable for the parameter sweep associated with amplitude *A* (Fig. 2, top row). The average distance between simulated strains grows gradually, and often monotonically, from approximately 0.04 (min) to 0.17 (max) as we look at strains located further away from each other. Physically, distinctions in motility are more obvious between strains with extremely low and high body amplitudes.

For the other two simulated datasets (Fig. 2, middle and bottom rows), the gradual pattern also exists but becomes more subtle and complex, where maximal values in the average distances observed are smaller (approximately 0.11 and 0.13 for frequency and wavenumber, respectively). This follows partly from the tighter range of values simulated (Fig. 2, bottom row). Physically, nematodes display a wider range in amplitude changes compared to wavelength [30]; this may be a consequence of the finite range of gait modulation available to the organism through neuromuscular control [36, 37]. For example, looking back at Table 1, while the range of amplitudes in our simulations more than doubles from 16 to 35 pixels (Set I), the wavelength increases by less than 25% from 0.044 to 0.0535 (Set III). Thus, with a fixed 10% variance included for each simulated strain, the overlap between neighbouring strains increases in accordance with increasing parameter value; not surprisingly, the diagonals of the matrices illustrate small yet non-zero values. Nevertheless, the maximum standard error (S.E.) observed across all matrices remains less than 2.5% of the average distance.

In order to examine the significance of the differences obtained between any two pair of classes, we performed a non-parametric two-sample test for multivariate samples using the minimum statistical energy test described in [35], which is minimized when the two samples are drawn from the same parent distribution. Fig. 2 (middle column) presents the corresponding matrices of *p*-values obtained when comparing pairwise a given class with another. Note that matrices only show values in the upper-triangular region (due to symmetry) where the diagonal is not computed; significance (black tiles) is set for a confidence level of 95% (*p* ≤ 0.05). Our results indicate that statistical significance is overwhelmingly met for the datasets sweeping through both amplitude (top row) and frequency (middle row) changes, whereas for changes in wavelength (bottom row) results are significant for ∼ 50% of the cases. This latter result is not surprising given the tight parameter sweep (noted above) and hence the large relative overlap given a fixed 10% variance. As such, our algorithm is most sensitive to amplitude and frequency changes over the physiological range of parameters simulated under crawling conditions [30].

Corresponding dendrograms (Fig. 2, right column) for the distance matrices are computed according to the well-established numerical classification scheme described in [38]. Briefly, this branching diagram represents a hierarchy based on the relationships of similarity among different classes. The algorithm is based on (i) first computing Euclidean distances between classes (as seen in Fig. 2, left column), (ii) grouping pairs of classes into a binary, hierarchical cluster tree according to the distances and, (iii) finally pruning branches off the bottom of the hierarchical tree, and assign all the classes below each cut to a single cluster so as to partition the data; this last step is done by detecting natural groupings in the hierarchical tree. Ideally, for our synthetic datasets one would expect a gradual and monotonic ordering across the classes (sequentially from 1 to 20). Our results generally capture such sequencing, although a number of permutations in the ordering of classes arises and breaks the monotonic trend. As anticipated, such permutations are most pronounced for the parameter sweep in *k* (bottom row). Nevertheless, the clustering of classes remains generally successful and overall our algorithm is capable of discriminating between classes according to the (average) Euclidean distance between histograms of visual words.

### Nematode motility data I

**Fig 3:**
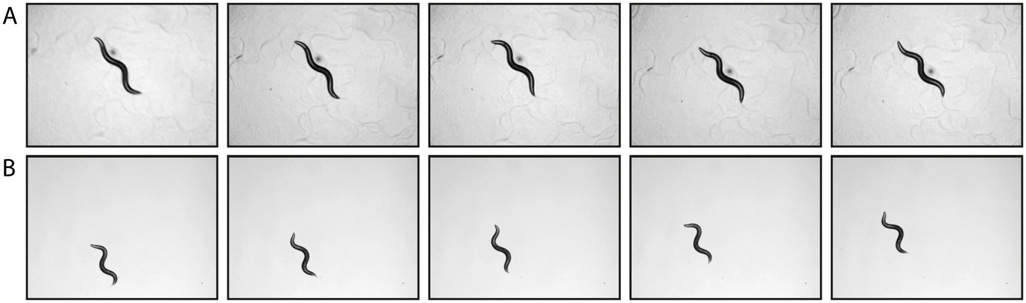
Instantaneous frames of nematode crawling assays. (A) Sequence showing a crawling nematode kept in the center of the field of view (FOV) using a motorized stage and custom software that automatically tracks the worm; data taken from [25]; see Results section for “Nematode motility data I”. (B) Sequence showing a nematode crawling in a fixed FOV; see Results section for “Nematode motility data II”. In both (A) and (B), time-lapse sequence shown for every 5th frame and nematodes are approximately 1 mm long.

Following the above results, we then analysed a subset of videos from a previously published worm behaviour database [25]. Briefly, each raw video spans 15 minutes and contains a single young adult hermaphrodite that is spontaneously behaving on a patch of bacterial food (*E. coli*). Nematodes are kept in the center of the field of view (FOV) using a motorized stage and custom software that automatically tracks the worm (Fig. 3A); see Yemini *et al*. [25] for a more complete description of the methodology.

For each strain investigated, 20 videos per strain were randomly selected for analysis. The camera magnification was set between 3.5 and 4.5 *μ*m per pixel, corresponding to a FOV of approximately 2.5 × 2 mm^2^ at 640 × 480 pixel resolution. The frame rate was set at 20–30 fps. Next, video frames were down-sampled by a factor of 5 (i.e. sampling every 5th frame) in order to decrease the video size as well as increase the difference in nematode displacement between consecutive frames. Each frame was then segmented using a simple thresholding step to separate worms from the background [23].

We tested our algorithm on 15 strains, where we included several groups of strains based primarily on their genetic relationships. The first subset contains strains that have different mutations affecting the same gene (*egl-21(n611)* and *egl-21(n476)*, *trpa-2(tm3085)*, *trpa-2(tm3092)* and *trpa-2(ok3189)*, *unc-98(st85)* and *unc-98(su130)*, *unc-108(n777)* and *unc-108(n501)*). The second subset has mutations in genes that code for different parts of known protein complexes (*unc-63(ok1070)* and *unc-38(e264)*, *unc-79(e1068)* and *unc-80(e1069)*). Finally, we also included two strains that were found to cluster together in a previous analysis (*acd-5(ok2657)* and *asic-2(ok289)*) [23]. Note that unlike our simulated datasets, videos of real worms (this also includes “Nematode motility data II”, see below) are not restricted to capturing forward motion only but sample rather a broad range of locomotion traits (*e.g*. pauses, backward motion, etc.); moreover, mutant strains may potentially exhibit locomotory behaviours that are not seen in wild type nematodes, including rolling, coiling, omega bends, to name a few.

**Fig 4:**
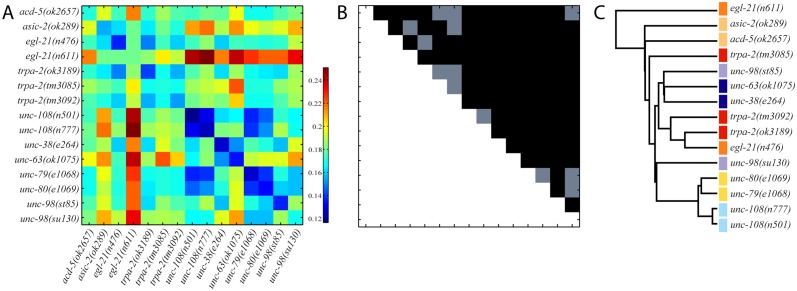
Locomotion similarity between 15 mutant strains. (A) Distance matrix of average Euclidean distances between mutant strains (strains are ordered alphabetically). (B) Corresponding matrix for *p*-values for pairwise strain comparisons. Black tiles indicate significant differences, *p* < 0.05 using the minimum energy test. (C) Hierarchical clustering (dendrogram) of mutant strains based on the distance matrix in A. The coloured bars indicate strains that are expected to cluster together based on known functional relationships or, in the case of *acd-5* and *asic-2*, previous clustering results from other methods [23].

Average distances between strains are presented in Fig. 4A and range between approximately 0.11 (min) and 0.25 (max), with a maximum standard error less than 2.8% of the average distance. Following the clustering method described above, the corresponding clustering tree is shown in Fig. 4C. We find that 3 out of 7 expected groups are clustered as nearest neighbours with 2 other pairs separated by only one branch. In pairwise comparisons, we find statistically significant differences for 92 out of 105 strain pairs (Fig. 4B). Furthermore, out of the 13 pairs where the difference was not significant, 5 belonged to genetically related groups; for example, looking at Fig. 4B, cells {5, 6}, {5, 7} and {6, 7} do not exhibit significant differences between the strains with different alleles of *trpa-2*.

In other words, the algorithm is able to detect differences between almost all of the strain pairs while still capturing similarities in locomotory phenotypes that lead to a clustering that is consistent with expectations based on genetic relationships between strains. This is the case even though our approach was conducted without having any worm-specific features defined. We briefly note that collection of worm data was randomized; as such, day-to-day effects are not anticipated in the outcome of our phenotyping analysis and strains that cluster together do not correlate with recordings obtained at similar dates and/or times [25]. As a final note, the total computational runtime (*i.e*. 15 strains × 20 videos/strain × 900 frames/video = 270,000 frames of 640 × 480 pixel size) using 12 cores is approximately 196 min (5 independent runs), and is partitioned between building the visual vocabulary (43 min) and generating the corresponding histograms (153 min).

### Nematode motility data II

To further assess the performance of our SIFT-BoW algorithm, we next tested our approach with several mutant strains affecting neuromuscular structure and function from a previously published dataset [22]. Briefly, video acquisition was performed on hypochlorite-synchronized young adult animals grown at 25^*o*^C (Fig. 3B). Worms were transferred to 3cm NGM plates with no food for 2 minutes before recording. Recordings of *C. elegans* crawling (approximately 10 s long) were obtained by a Leica S8APO microscope equipped with a Leica DFC 295 camera (1024 × 768 pixels) at 26 fps using standard bright field microscopy at 32× magnification. For each strain investigated (i.e. all videos are available at http://dx.doi.org/10.7910/DVN/29216), 10 videos per strain were randomly selected for analysis; as described above, video frames were down-sampled by a factor of 5 (*i.e*. sampling every 5th frame) and segmented using a simple thresholding step to separate worms from the background. The following strains were used: N2(wild-type); *dys-1(ls292)*; *dyb-1(ls505)*; *unc-17(cb933)*; *acr-16(rb918)*; *acr-2(rb1559)*; *sgn-1(rb1882)*; *ace-1(vc505)*. All *C. elegans* strains were obtained from the Caenorhabditis elegans Genetic Stock Center (CGC) and maintained using standard culture methods [2]. Note that the aforementioned strains include well-described mutants affecting two aspects of neuromuscular function: synaptic transmission and sarcomere stability.

**Fig 5:**
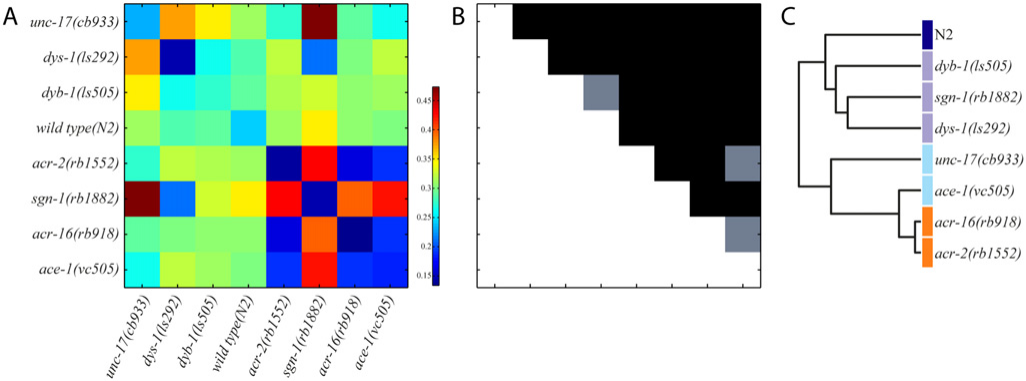
Locomotion similarity between 8 mutant strains. (A) Distance matrix of average Euclidean distances between mutant strains (strains are ordered alphabetically). (B) Corresponding matrix for *p*-values for pairwise strain comparisons. Black indicates significant differences, *p* < 0.05 using the minimum energy test. (C) Hierarchical clustering (dendrogram) of mutant strains based on the distance matrix in A.

In Fig. 5 we present results for (A) the pairwise inter-distance similarity matrix, (B) the corresponding significance test, and (C) the resulting clustering tree (dendrogram). We note that the average pairwise distances between strains range from approximately 0.13 (min) up to 0.47 (max), with a maximum standard error (S.E.) less than 6.7% of the average distance. In pairwise comparisons, we find significant differences for 53 out of 56 pairs (Fig. 5B).

As expected, our computational clustering method reveals that genes with related biological function cluster closest together (Fig. 5C). For example, *dys-1* mutants robustly cluster with *sgn-1* and *dyb-1* mutants. Indeed, *dys-1* (dystrophin), *sgn-1* (sarcoglycan), and *dyb-1* (dystrobrevin) encode protein components of the dystrophin-associated glycoprotein complex (DAGC), which links the cytoskeleton to the extracellular matrix in muscles [39]. Similarly, the other cluster is primarily composed of genes involved in acetylcholine signaling, such as *acr-2* and *acr-16* (nicotinic acetylcholine receptor subunits) [40, 41], *ace-1* (acetylcholinesterase) [42], and *unc-17* (synaptic vesicle acetylcholine transporter) [43]. Altogether, these results further support the utility of our approach.

As a final note, the total computational runtime (*i.e*. 8 strains × 10 videos/strain × 70 frames/video = 5,600 frames of 1024 × 768 pixel size) using 12 cores is approximately 7.3 min (5 independent runs), and is partitioned between building the visual vocabulary (1.7 min) and generating the corresponding histograms (5.6 min).

## Discussion and Outlook

In the present work, we have attempted to develop a robust, relatively fast and fully-automated phenotyping algorithmic approach to evaluate similarity relations between different strains of *C. elegans*, with no need for defining nematode-specific morphological or kinematic features. While widely-accepted “ground truth” data regarding proximity relations between nematode strains are still not widely available, we have shown in the examples above that our approach delivers clustering results on real nematode data that is coherent with other nematode-specific feature-based techniques [22, 23, 25].

Overall, SIFT descriptors are found to be computationally efficient when attempting to describe nematode posture and locomotion. By applying such descriptors not only to individual static frames but differences between frames as well, we were able to extract information associated with nematode posture (*i.e*. amplitude, wavelength), body frequency and locomotion speed amongst others. We achieved reasonable performance using SIFT descriptors but it may be possible to improve the method in the future using other kinds of computer vision-based descriptors, including for example Shape Context descriptors [44], Self-Similarity descriptors [45] or Global Self-Similarity descriptors [46].

We note that for the crawling assays tested, the segmentation scheme (*i.e*. simple thresholding technique) we used was simple to implement and relatively fast. However, on other types of nematode data, such as with lower image quality or featuring other motility assays (*e.g*. swimming assays, microfluidic cells, etc.), a simple thresholding scheme is anticipated to perform significantly less well [47]. For such applications, more elaborate segmentation strategies relying for example on the use of combined intensity and texture-based features integrated within a probabilistic framework [48] have shown great promise and can easily be integrated into our current approach.

It must be stressed again that one underlying limitation of our approach lies in that the resultant relations between strains cannot be straightforwardly translated back into morphological or kinematic characteristics of locomotion. Unlike alternative methods that rely on defining and extracting physical characteristics, the physical origin for the distance metric obtained between strains may not be easily deduced. Unlike methods that require the pre-determination of physical metrics of interest [10, 11, 14], the user is only required here to arrange the videos into folders and the algorithm then proceeds automatically. The outlined approach is in this sense a fully-automated motility phenotyping scheme.

As a final note, the scheme presented here is very general in nature and can be applied in principle to other tasks relating to motility phenotyping, with small local modifications. Such tasks could include investigating different aspects of *C. elegans* biology including development stages or sex (males vs. hermaphrodites) or expanding possibly to other model organisms (*e.g*. flies, fish or mice). Such applicability across other animal models will be particularly useful as more groups collect high-resolution image data of animal behaviour [31].

## Methods

**Fig 6:**
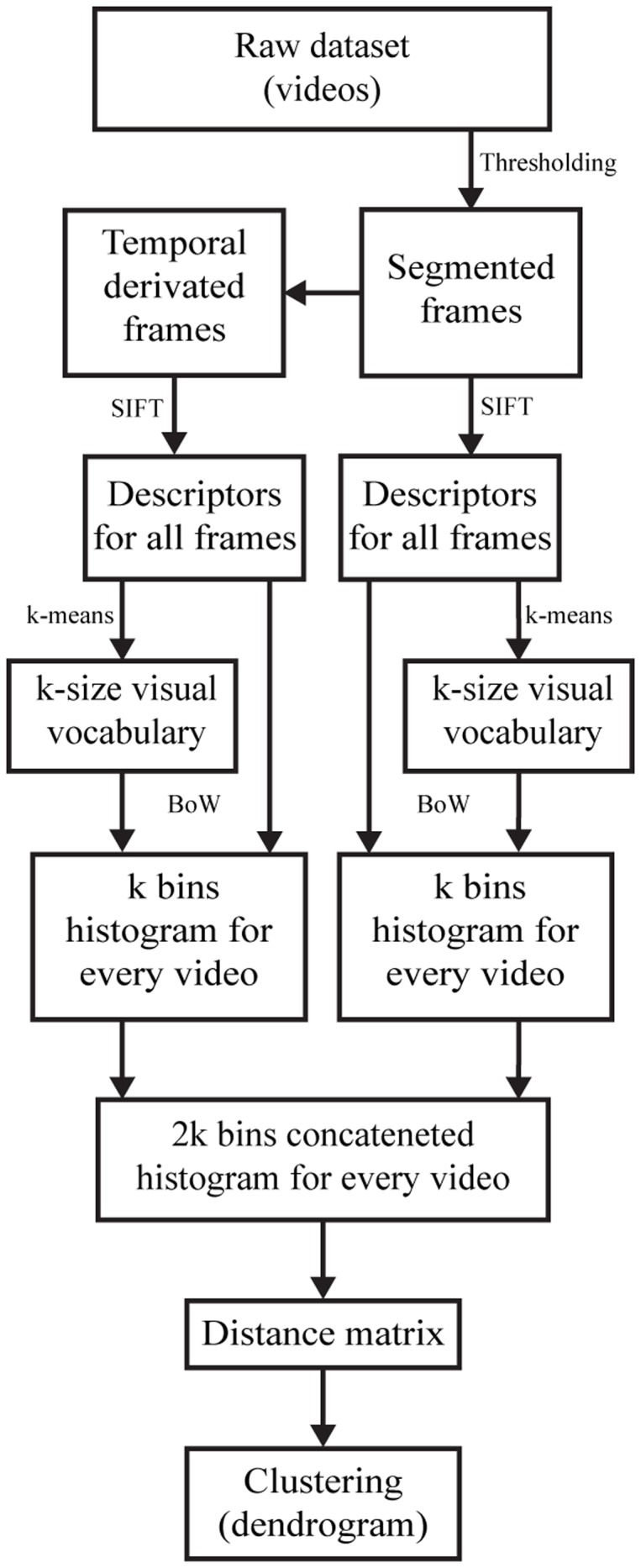
Schematic flowchart of the SIFT-BoW algorithm. See Methods section for details.

Our method relies on the widely-used Bag-of-Words (BoW) model [49, 50] to represent motility videos as histograms of typically occurring Scale-Invariant Feature Transform (SIFT) descriptors [51]. Our implementation is a variation of the SIFT-bag model introduced in [52]. The algorithm was implemented in Matlab and relies on the freely available VL_FEAT library [53]. The algorithm is schematically illustrated in Fig. 6.

### Data organization and preprocessing

Each video is preprocessed to simplify analysis. We begin by automatically segmenting the nematode from its background by applying a fixed threshold to the image pixels and do so on each frame of a given video in the dataset; note that automatic segmentation of nematodes in more complex environments can also be achieved by using more sophisticated strategies as recently detailed in Greenblum *et al*. [48].

In addition, we pre-sampled the volumes every 5 frames in order to reduce the quantity of data and also to increase the variability between consecutive frames. The pre-sampled segmented volumes were used for further analysis.

### Building a visual vocabulary

From the complete set of videos, we randomly select a quarter of the videos, spread out uniformly across each nematode class. This small subset will be used to generate a visual vocabulary which will allow us to visually characterize motility. From each video of the subset, we randomly select *N* = 15 blocks of *M* = 60 consecutive frames making sure that none of the selected blocks overlap. Note that for very short videos, this random block sampling was omitted. From these blocks and using the segmented images from the preprocessing stage, SIFT keypoints were computed on nematode pixels only and associated descriptors were extracted.

We then clustered each of the SIFT descriptors extracted from the blocks using a *k*-means clustering algorithm (not to be confused with the wavenumber *k*, see Table 1). This clustering yields *k* canonical, or *mean* descriptors, such that any descriptor can be categorized as belonging to one of the *k* mean descriptors by computing the minimum Euclidean distance to each mean descriptor.

Using another randomly selected 25% of videos (with overlap) we repeat the process described in this subsection this time computing the SIFT keypoints and descriptors not on the original image data but rather on the temporal derivate images. These temporal images are computed by subtracting consecutive frames of the original data. Hence, this provides motion information over time. From these new SIFT descriptors, we compute a new set of *k* mean descriptors using *k*-means clustering again.

Together, both set of *k* mean descriptors (one on the raw image data and one on the temporal gradient image data) characterize our visual vocabulary. The Davies-Bouldin index [54] was used for the optimization of *k*. The vocabulary is stored in a k-d tree structure [55] in order to ease the search for closest match while building the histograms in the following step.

### Video characterization by histograms

Using the nematode segmentations, we compute SIFT descriptors in *N* non-overlapping blocks of *M* frames on both the raw image and the temporal images (computed as described above).

The collected SIFT descriptors from the segmented images are then used to build a *k*-sized histogram, *i.e*. *k* bins. For each SIFT descriptor extracted, we compute its Euclidean distance to each of the mean descriptors. We attribute to a particular SIFT descriptor the mean descriptor which is closest to it in terms of Euclidean distance. Having done so for each SIFT descriptor, we count the number of occurrences of each mean descriptor in the selected blocks, which results in a histogram of size *k* bins. To preserve invariance to the overall number of descriptors collected, the histogram is normalized to sum to one. The same operations are then repeated for the temporal difference image SIFT and mean descriptors, yielding a second *k* size histogram. Both histograms are then concatenated together to form a 2*k* sized histogram. This process can then be performed on each video in the dataset.

### Calculating a distance matrix

Having characterized videos by an informative histogram, we now build a distance matrix over the entire dataset. Each cell in the matrix represents the average distance between two classes. Examples of such distance matrices are shown in the results of Figs. 2, 4 and 5. Such maps visually depict the distance matrix and naturally, the matrix is symmetric along its diagonal. In each element of the matrix, we depict the mean distance between two classes which we compute as being the mean Euclidean distance between each possible pairing of both classes, *e.g*. two classes with 20 videos would yield 20 × 20 = 400 possible distances; note that, the resulting distance between two volumes represents the Euclidean distance between their two respective histograms and thus has arbitrary units. From such data, we can thus also compute the average ± standard error (S.E.) of the pairwaise inter-distances between classes.

## Supporting Information

S1 Script Open source Matlab script package for the SIFT-BOW algorithm. (ZIP)

S2 Script Matlab script to generate sequences of synthetic worms according to beating frequency, wavelength and body amplitude. (M)

S1 Video Example of synthetic worm crawling (*A* = 16 [pixel], *f* = 0.36 [Hz], *k* = 0.05 [1/pixel]). (AVI)

S2 Video Example of synthetic worm crawling (*A* = 35 [pixel], *f* = 0.36 [Hz], *k* = 0.05 [1/pixel]). (AVI)

S3 Video Example of synthetic worm crawling (*A* = 18 [pixel], *f* = 0.06 [Hz], *k* = 0.05 [1/pixel]). (AVI)

S4 Video Example of synthetic worm crawling (*A* = 18 [pixel], *f* = 0.44 [Hz], *k* = 0.05 [1/pixel]). (AVI)

S5 Video Example of synthetic worm crawling (*A* = 18 [pixel], *f* = 0.36 [Hz], *k* = 0.44 [1/pixel]). (AVI)

S6 Video Example of synthetic worm crawling (*A* = 18 [pixel], *f* = 0.36 [Hz], *k* = 0.0535 [1/pixel]). (AVI)
